# Molecular determinants of the selectivity and potency of α-conotoxin Vc1.1 for human nicotinic acetylcholine receptors

**DOI:** 10.1016/j.jbc.2024.108017

**Published:** 2024-11-26

**Authors:** Han-Shen Tae, Andrew Hung, Richard J. Clark, David J. Adams

**Affiliations:** 1 Molecular Horizons, Faculty of Science, Medicine and Health, University of Wollongong, Wollongong, Australia; 2 School of Science, RMIT University, Melbourne, Australia; 3 School of Biomedical Sciences, The University of Queensland, Brisbane, Australia

**Keywords:** α-conotoxin, electrophysiology, molecular dynamics, nicotinic acetylcholine receptors (nAChR), site-directed mutagenesis, *Xenopus* oocytes

## Abstract

The α-conotoxins (α-Ctxs) are short, disulfide-rich peptides derived from the venom of the *Conus* marine snails, primarily acting as antagonists of nicotinic acetylcholine receptors (nAChRs). Specifically, α-Ctx Vc1.1, a 16-amino acid peptide from *Conus victoriae*, competitively antagonizes non-muscle nAChRs, inhibits nicotine-induced currents in bovine chromaffin cells, and alleviates neuropathic pain in rat models. Although Vc1.1 selectively inhibits rat α9α10 nAChRs, its potency and selectivity across human nAChR subtypes remain unresolved. In this study, we assessed the activity of Vc1.1 on human (h) nAChRs heterologously expressed in *Xenopus laevis* oocytes using the two-electrode voltage clamp technique and simulated interactions using computational modeling. Vc1.1 selectively antagonized homomeric α9 and heteromeric α3β2 nAChRs, with half-maximal inhibitory concentrations (IC_50_) of 160 nM and 232 nM, respectively. At hα9[N179A]α10, Vc1.1 exhibited a 20-fold decrease in potency compared to hα9α10, due to the loss of hydrogen bonding with Vc1.1-D11. Conversely, Vc1.1 was four-fold more potent at hα3β2[E86A] compared to hα3β2, possibly influenced by the proximal residue β2-K104, as suggested by molecular dynamics (MD) simulations. Additionally, Vc1.1’s potency doubled at hα9[N213K]α10, whereas it remained unchanged at hα9[N213R]α10 nAChRs. MD simulations indicate that altered interactions between the mutant hα9 N179A, N213K, and N213R side chains and Vc1.1-D5 may partly explain these changes in potency. The inhibitory action of Vc1.1 at α9-containing nAChRs is particularly relevant given their role in neuroinflammation, presenting a potential therapeutic pathway for alleviating neuropathic and inflammatory pain. This study provides valuable insights into the rational design of Vc1.1-derived α-Ctxs with enhanced nAChR subtype selectivity.

Conotoxins are disulfide-rich peptides found in the venom of *Conus* snails, with the α conotoxin (α-Ctx) peptide family primarily acting as competitive antagonists of muscle and neuronal nicotinic acetylcholine receptors (nAChRs). These receptors are pentameric ligand-gated ion channels, and to date, 17 different nAChR subunits have been identified (α1-α10, β1-β4, γ, δ, and ε). nAChRs mediate cholinergic synaptic transmission at the neuromuscular junction and within both the central and the peripheral nervous systems (1, 2, 3). In addition to their roles in neurological disorders such as addiction, depression, epilepsy, attention-deficit hyperactivity disorder (ADHD), schizophrenia, Alzheimer’s disease, Parkinson’s disease, and chronic pain (3), nAChRs are also implicated in the carcinogenesis of certain cancers (4, 5, 6) and in the regulation of the immune system (7, 8).

α-Conotoxin Vc1.1 is a C-terminal amidated peptide consisting of 16 amino acids, stabilized by two disulfide bridges in a globular (CysI-CysIII, CysII-CysIV) conformation (Fig. 1). It was initially identified through transcriptomic analysis of the molluscivorous *Conus victoriae* collected from Broome, Western Australia (9). Vc1.1 belongs to the α-Ctx peptide family and is known to target neuronal nAChRs (10). Synthetic Vc1.1 acts as a competitive antagonist in nicotine-induced catecholamine release assays and inhibits nicotine-evoked currents in cultured bovine chromaffin cells, with half-maximal inhibitory concentration (IC_50_) of 1 to 3 μM (9, 10). The peptide does not inhibit potassium-induced catecholamine release (9). Notably, Vc1.1 displaced the nicotinic receptor agonist epibatidine in binding assays on bovine adrenomedullary membranes, confirming that its molecular targets are nAChRs rather than voltage-gated ion channels (9). Furthermore, Vc1.1 effectively relieved pain in several rat models of neuropathic pain, with efficacy lasting up to 24 h post-administration at higher doses in the rat chronic constriction injury (CCI) model (11).

**Figure 1:**
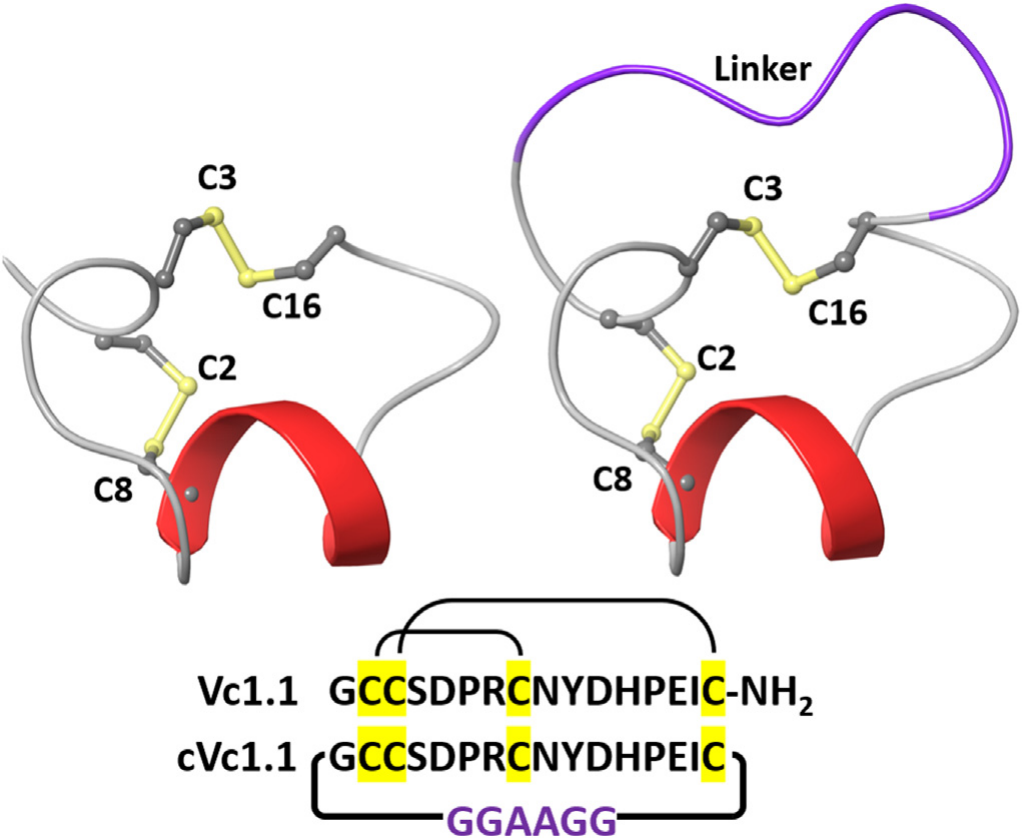
**Structure and amino acid sequences of α-conotoxins Vc1.1 and cyclic Vc1.1 (cVc1.1).** In these sequences, the cysteine residues are depicted as small spheres, with the sulphur atoms in the disulfide bonds highlighted in *yellow*. For cVc1.1, a *purple* linker sequence (GGAAGG) connects the amino and carboxy termini, creating a cyclic form.

Vc1.1 has been shown to selectively inhibit acetylcholine (ACh)-evoked currents mediated by rat α9α10 nAChRs heterologously expressed in *Xenopus laevis* oocytes, with an IC_50_ ranging from 19 to 109 nM (12, 13, 14, 15). However, its potency at the human α9α10 nAChR was lower, with an IC_50_ ranging from 320 to 1000 nM (16, 17). Despite this, the selectivity of Vc1.1 for other human nAChR subtypes has not been thoroughly investigated. To our knowledge, the only other human subtype tested was the α7 nAChR, for which the IC_50_ was greater than 10 μM (17). Therefore, this study was conducted to evaluate the selectivity and potency of α-Ctx Vc1.1 across various human nAChR subtypes, heterologously expressed in *X. laevis* oocytes and examined using the two-electrode voltage clamp technique. Results revealed that Vc1.1 is a selective antagonist for the α3β2 and α9 nAChR subtypes. Additionally, we investigated the effects of specific nAChR amino acid residue mutations on activation by ACh and inhibition by Vc1.1, providing insights into the roles of these residues in nAChR function. Through molecular dynamics (MD) simulations, we also identified potential molecular determinants responsible for Vc1.1’s selective interactions with human α3-and α9-containing nAChR subtypes.

## Results

### Vc1.1 preferentially antagonizes heterologous human **α**3**β**2, **α**6∗-, and **α**9-containing nAChR subtypes

**Figure 2:**
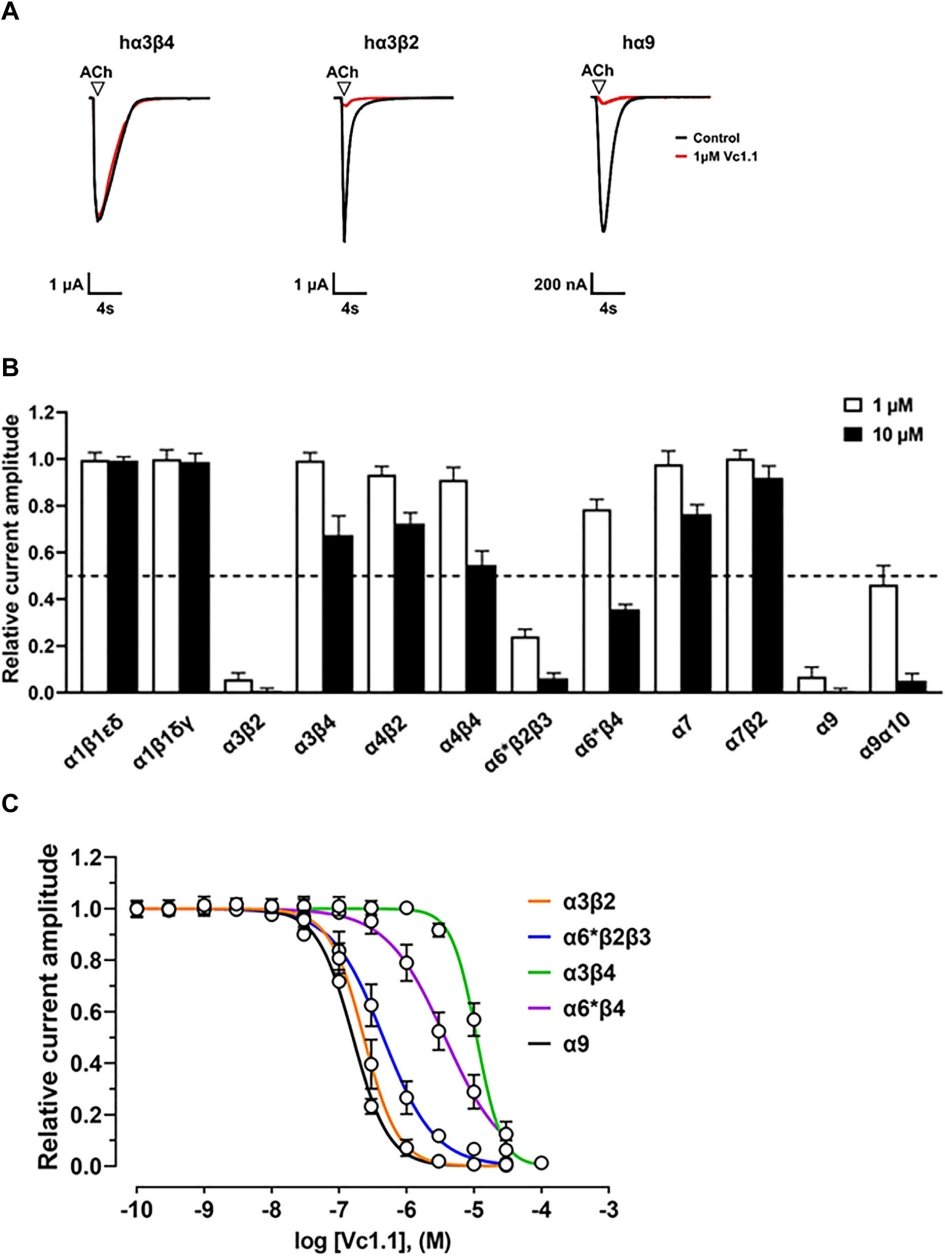
**Activity of α-conotoxin Vc1.1 at the human nAChR subtypes.***A*, representative ACh (300 μM, 6 μM, and 50 μM, respectively)-evoked currents mediated by hα3β4, hα3β2, and hα9 nAChRs in the absence (*black*) and presence (*red*) of 1 μM Vc1.1. *B*, bar graph of the inhibition of ACh-evoked peak current amplitude by Vc1.1 (at 1 and 10 μM) across various heterologous human nAChR subtypes (mean ± SD, n = 5–10). The dashed line indicates 50% inhibition of current amplitude. *C*, concentration-response relationships of relative ACh-evoked current amplitude mediated by hα3β2, hα3β4, hα6∗β2β3, hα6∗β4, and hα9 nAChRs in the presence of α-Ctx Vc1.1 (mean ± SD, n = 5–12). The calculated IC_50_ and n_H_ values are summarized in Table 1.

At a concentration of 1 μM, α-Ctx Vc1.1 reversibly inhibited less than 10% of the ACh-evoked current amplitude mediated by the following nAChR subtypes: hα1β1εδ, hα1β1δγ, hα3β4, hα4β2, hα4β4, hα7, and hα7β2 (n = 5–10) (Fig. 2, *A* and *B*). In contrast, the hα3β2 and hα9 nAChR subtypes were inhibited by more than 95% (Fig. 2*A*). The hα6∗β2β3 (∗α6/α3 chimera) was inhibited by 75%, the hα9α10 subtype by 50%, and the hα6∗β4 subtype by ∼30%.

At a concentration of 10 μM, Vc1.1 inhibited all nAChR subtypes except for both muscle nAChR subtypes (n = 5–9). Vc1.1 completely inhibited the ACh-evoked currents mediated by α3β2 and α9 subtypes and inhibited >90% of the ACh-evoked current amplitude for the α6∗β2β3 and α9α10 subtypes. In comparison, the α6∗β4 subtype was inhibited by ∼60%, whereas the α3β4, α4β2, α4β4, and α7 subtypes were inhibited by about 30 to 45%. In contrast, α7β2 nAChRs were inhibited by <10%. Thus, the selectivity profile of Vc1.1 across the human nAChR subtypes tested is as follows: α3β2 and α9 > α6∗β2β3 and α9α10 > α6∗β4 > α4β4 > α3β4, α4β2, and α7 > α7β2 > α1β1εδ and α1β1δγ.

Concentration-response relationships showed that Vc1.1 had comparable potencies at hα3β2 and hα9 nAChRs with IC_50_ values of 232.5 nM (220.3–245.1: 95% CI, n = 8) and 160.4 nM (148.5–173.5: 95% CI, n = 5–8), respectively (Fig. 2*C*, Table 1). This was followed by the α6∗β2β3 and α9α10 subtypes with IC_50_ values of 451.2 nM (420.1–484.6: 95% CI, n = 9–12) and 906.9 nM (847.0–971.1: 95% CI, n = 6–8), respectively. Additionally, at β4-containing nAChRs, Vc1.1 was less potent at α3β4 nAChRs with an IC_50_ of 10.95 μM (10.55–11.37: 95% CI, n = 10–12), and the α6∗β4 subtype with an IC_50_ of 3.72 μM (3.44–4.02: 95% CI, n = 9) (Fig. 2*C*, Table 1).

**Table 1:** Pharmacological activity of **α**-conotoxin Vc1.1 and cyclized Vc1.1 (cVc1.1) at human nAChR subtypes

nAChR	Vc1.1		cVc1.1	
	a IC_50_ (nM; 95% CI)	n_H_ (95% CI)	a IC_50_ (nM; 95% CI)	n_H_ (95% CI)
α3β2	232.4 (220.3–245.1) [8]	1.7 (1.6–1.8) [8]	217.1 (209.4–225.1) [12]	2.2 (2.1–2.4) [12]
α3β4	10,950.0 (10,550.0–11,370.0) [10–12]	2.3 (2.1–2.6) [10–12]	11,060.0 (10,450.0–11,700.0) [9–11]	1.7 (1.5–1.9) [9–11]
α6∗β2β3	451.2 (420.1–484.6) [ 9–12]	1.1 (1.1–1.2) [9–12]	ND	
α6∗β4	3719.0 (3439.0–4022.0) [9]	1.0 (0.9–1.1) [9]	ND	
α9	160.4 (148.5–173.5) [5–8]	1.6 (1.5–1.7) [5–8]	163.1 (153.6–173.1) [11–12]	1.2 (1.2–1.3) [11–12]
α9α10	906.9 (847.0–971.1) [6–8]	1.5 (1.4–1.7) [6–8]	1196.0 (1152.0–1241.0) [11]	2.0 (1.9–2.2) [11]

Number of oocytes in square brackets.

IC_50_, half-maximal inhibitory concentration; n_H_, Hill coefficient; ND, not determined.

a Mean ± 95% CI (confidence interval).

Concentration-response relationships were also determined for a cyclic version of Vc1.1, cVc1.1 (Fig. 1) (18), at hα3β2, hα3β4, hα9, and hα9α10 yielding IC_50_ values of 217.1 nM (209.4–225.1: 95% CI, n = 12), 11.06 μM (10.45–11.70: 95% CI, n = 9–11), 163.1 nM (153.6–173.1: 95% CI, n = 11–12), and 1196.0 nM (1152.0–1241.0: 95% CI, n = 11), respectively. The IC_50_ values obtained for cVc1.1 at each nAChR subtype were similar to those obtained for Vc1.1 (Figs. S1–3 and Table 1).

### Homology models of Vc1.1-bound h**α**3**β**2 and h**α**9**α**10 nAChRs and selection of mutation sites

**Figure 3:**
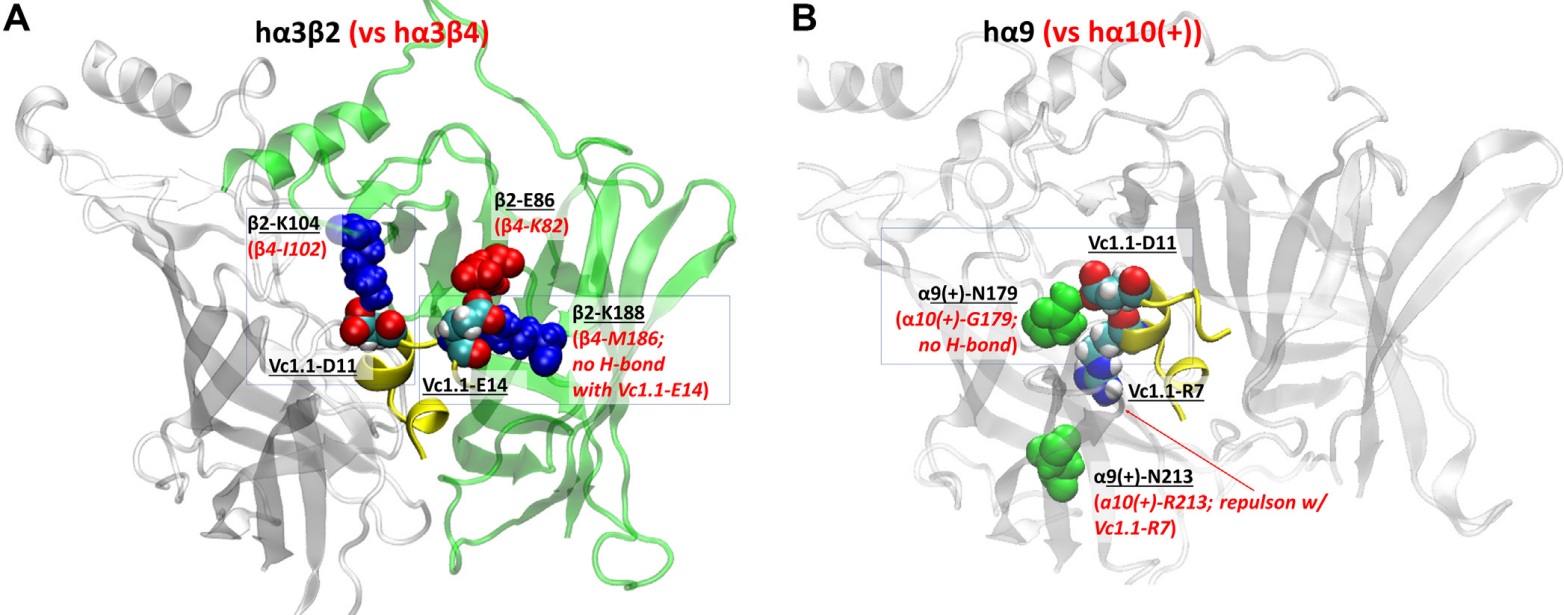
**Homologymodel of α-conotoxin Vc1.1bound to theextracellular domainof nAChRs.***A,* ribbon structure of hα3β2 (hα3 in *white*, β2 in *green*) bound to Vc1.1 (*yellow ribbon*), highlighting key differences with hα3β4. *B,* hα9 bound to Vc1.1, highlighting key differences between the hα9(+) and hα10(+) subunits.

In hα3-containing nAChR subtypes, two positions on the β subunits, E86 and K188 in β2, are of particular interest. These residues differ between β2 and β4 and are both located near the α-Ctx binding site, where they can form salt bridge interactions with Vc1.1. Homology modeling revealed that Vc1.1-E14 is located close to β2-E86 (Fig. 3*A*), suggesting mutual electrostatic repulsion, whereas the equivalent position in β4 is K82. This finding suggests that neutralizing or reversing the charge at β2-E86 could alter the receptor’s sensitivity to Vc1.1. Additionally, an interaction between Vc1.1-E14 and β2-K188 indicates that mutating this site may also affect Vc1.1 potency, as suggested by both homology modeling and AlphaFold3 predictions (Fig. S4).

At the hα9α10 nAChR, homology modeling and AlphaFold3 prediction (Fig. S5) indicate that Vc1.1-D11 forms a hydrogen bonding interaction with α9(+)-N179, an interaction that is absent at the homologous α10(+)-G154 position (Fig. 3*B*). Initial homology models also suggest that the α9(+)-N213 position might partly explain the difference in Vc1.1 activity between homomeric hα9 and heteromeric hα9α10 nAChRs. In the α10 subunit, the equivalent residue is R213, which could create electrostatic repulsion with Vc1.1-R7, potentially reducing Vc1.1 binding affinity. In contrast, no such repulsion would occur at the homologous α9(+)-N213, potentially enhancing the potency of Vc1.1 at hα9 compared to hα9α10.

### Activity of acetylcholine at wild-type and mutant h**α**3**β**2 and h**α**9**α**10 nAChRs

Homology modeling of Vc1.1 bound to the extracellular domain of human nAChRs postulated that the β2 subunit E86 and K188 residues, as well as the α9 subunit residues N179 and N213, are responsible for the differential inhibitory potencies of Vc1.1 at hα3β2 vs. hα3β4 and hα9 vs. hα9α10 nAChRs, respectively. To investigate this, alanine mutants of β2 E86 and K188, and α9 N179, as well as lysine/arginine mutants of α9 N213, were generated. The half-maximal effective concentration (EC_50_) values of ACh were determined for α3β2[E86A], α3β2[K188A], hα9[N179A]α10, hα9[N213K]α10, and hα9[N213R]α10 nAChRs.

**Figure 4:**
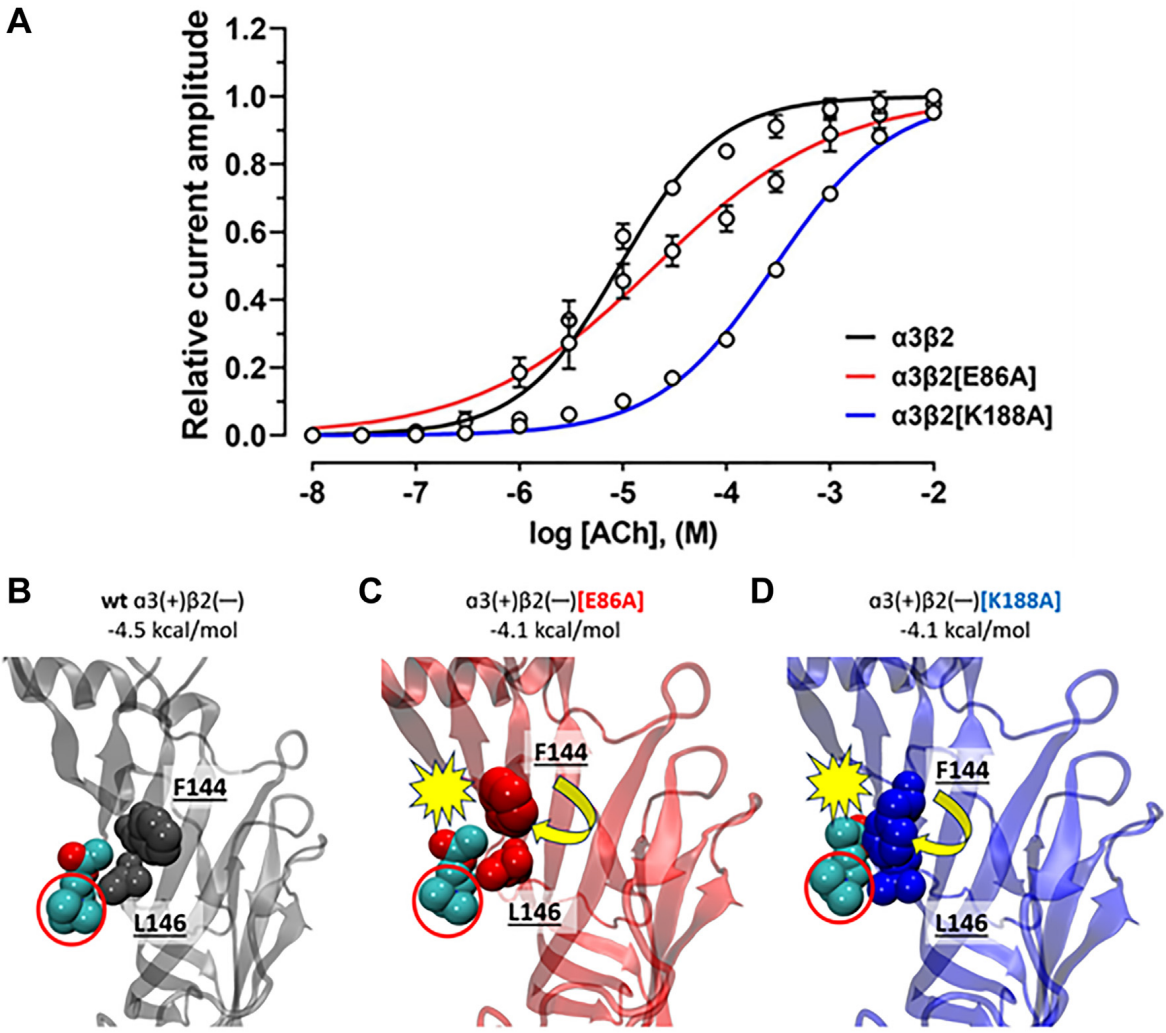
**Concentration-response relationships and molecular models of ACh activation of wild-type and mutant hα3β2 nAChRs.***A*, Concentration-response relationships of relative ACh-evoked current amplitude (mean ± SD, n = 3–9) mediated by hα3β2, hα 3[E86A]β2, and hα3[K188A]β2 nAChRs were measured across a concentration range of 10 nM to 10 mM ACh. The calculated EC_50_ and n_H_ values are summarized in Table 2. Docking poses for acetylcholine (*cyan* and *red* spheres) and its interactions with α10-F144 and L146 are shown for (*B*) wild-type hα3β2, (*C*) hα3β2[E86A], and (*D*) hα3β2[K188A]. *Yellow* spiked symbols indicate likely steric hindrance. Predicted binding affinity values are indicated for each. Only the β2(—) subunit is shown for clarity. The quaternary amine of ACh is circled in *red*.

**Figure 5:**
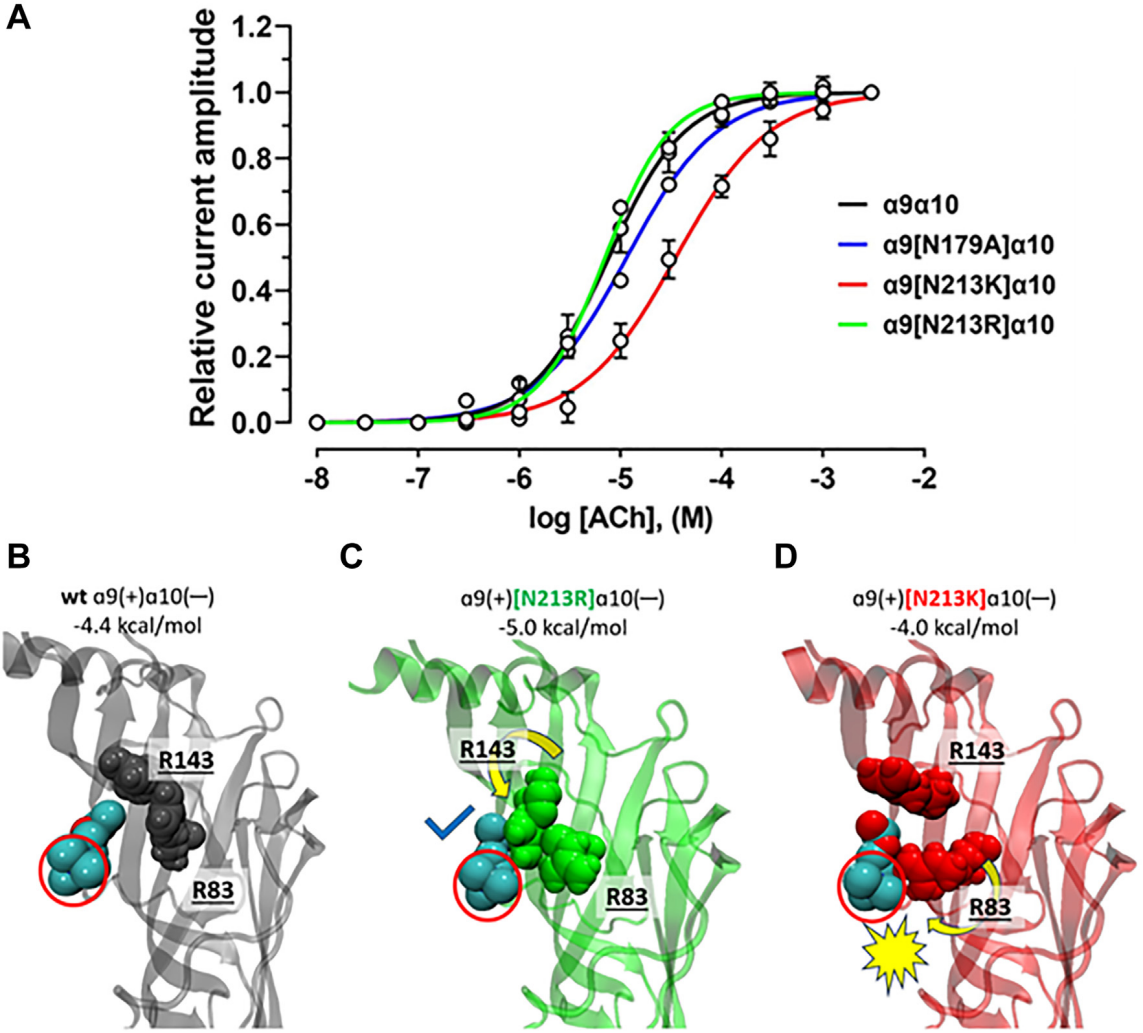
**Concentration-response relationships and molecular models of ACh activation of wild-type and mutant hα9α10 nAChRs.***A*, concentration-response relationships of relative ACh-evoked current amplitude (mean ± SD, n = 3–8) mediated by hα9α10, hα9[N179A]α10, hα9[N213K]α10, and hα9[N213R]α10 nAChRs were measured across a concentration range of 10 nM to 10 mM ACh. The calculated EC_50_ and n_H_ values are summarized in Table 2. Docking poses for acetylcholine (*cyan* and *red spheres*) and its interactions with α10-R83 and R143 are shown for (*A*) wild-type hα9α10, (*B*) hα9[N213K]α10 (tick symbol indicates favourable contact), and (*C*) hα9[N213R]α10 (*yellow* spiked symbol indicates likely steric hindrance), with predicted binding affinity values indicated for each. Only the α10(—) subunit is shown for clarity. The quaternary amine of ACh is circled in *red*.

Compared to α3β2 nAChRs, which have an ACh EC_50_ of 9.3 μM (8.0–10.8: 95% CI, n = 3), the α3β2[E86A] mutant showed an approximately two-fold decrease in sensitivity to ACh with an EC_50_ of 20.9 μM (18.1–24.3: 95% CI, n = 6–8) (*p* < 0.05). The β2[K188A] mutation resulted in a more than 30-fold decrease in ACh sensitivity, with an EC_50_ of 292.2 μM (268.8–317.7: 95% CI, n = 9) (*p* < 0.0001) (Fig. 4*A*, Table 2). Molecular docking predicted an increase (less negative) in binding energy for ACh relative to the wild-type α3β2 (−4.5 kcal/mol), for both α3β2[E86A] and α3β2[K188A] mutants (−4.1 kcal/mol) (Fig. 4, *B*–*D*). Although docking was not sensitive enough to reflect the differences in ACh activation potency observed experimentally between the two mutants, the homology models of the mutants showed minor structural differences in the F144 side chain. This side chain, which is distant from ACh in wild-type β2 (Fig. 4*B*), is rotated closer to ACh in both the E86A (Fig. 5*C*) and even more so in K188A (Fig. 4*D*). This closer rotation may underlie this K188A mutant’s relatively low sensitivity to ACh.

**Table 2:** Pharmacological activity of ACh and Vc1.1 at wild-type and mutant human nAChRs

nAChR	ACh		Vc1.1	
	EC_50_ (μM; 95% CI)	n_H_ (95% CI)	IC_50_ (nM; 95% CI)	n_H_ (95% CI)
α3β2	9.3 (8.0–10.8) [3]	0.9 (0.8–1.0) [3]	232.4 (220.3–245.1) [8]	1.7 (1.6–1.8) [8]
α3β2[E86A]	20.9 (18.1–24.3) [6–8]	0.51 (0.48–0.55) [6–8]	59.4 (56.5–62.4) [12]	1.8 (1.7–1.9) [12]
α3β2[K188A]	292.2 (268.8–317.7) [9]	0.76 (0.72–0.81) [9]	191.2 (184.1–198.7) [12]	1.9 (1.8–2.0) [12]
α9α10	7.6 (6.8–8.6) [3]	1.2 (1.0–1.3) [3]	906.9 (847.0–971.1) [6–8]	1.5 (1.4–1.7) [6–8]
α9[N179A]α10	11.5 (10.6–12.5) [8]	1.0 (0.9–1.1) [8]	17,920 (17,020–18,880) [7]	2.2 (2.0–2.4) [7]
α9[N213K]α10	35.0 (32.5–37.7) [7]	0.94 (0.88–1.00) [7]	489.4 (464.6–515.5) [10–11]	1.3 (1.2–1.4) [10–11]
α9[N213R]α10	7.0 (6.4–7.7) [6–7]	1.4 (1.2–1.5) [6–7]	897.4 (841.2–957.4) [9–10]	1.5 (1.4–1.6) [9–10]

EC_50_, half-maximal inhibitory concentration.

IC_50_, half-maximal effective concentration.

Number of oocytes in square brackets.

n_H_, Hill coefficient.

For the α9[N179A]α10 mutant, the EC_50_ for ACh was not significantly different (11.5 μM, 10.6–12.5: 95% CI, n = 8) compared to wild-type hα9α10 nAChRs (7.6 μM, 6.8–8.6: 95% CI, n = 3) (Fig. 5*A*, Table 2). In contrast, the α9[N213K] mutation caused a five-fold decrease in sensitivity to ACh (*p* < 0.0001) compared to the wild-type receptor, with an EC_50_ of 35 μM (32.5–37.7: 95% CI, n = 7). However, the ACh potency was unaffected by the α9[N213R] mutation, which had an EC_50_ of 7.0 μM (6.4–7.7: 95% CI, n = 6–7) (Fig. 5*A*, Table 2). Interestingly, the expression of each homomeric a9 mutant in oocytes did not produce a functional response to 1 mM ACh (n = 3).

Molecular docking of ACh to homology models of the agonist-bound conformations of wild-type and mutant hα9α10 nAChRs (Fig. 5, *B*–*D*) revealed a less negative predicted binding energy of ACh for α9[N213K] (−4.0 kcal/mol) compared to wild-type hα9α10 (−4.4 kcal/mol). Conversely, α9[N213R] showed a more negative binding energy for ACh (−5.0 kcal/mol), which moderately aligns with the slightly lower experimental EC_50_ value observed for this mutant. The models suggest that in wild-type hα9α10, the side chains of R83 and R143 line the edge of the binding pocket, allowing ACh to form favorable contacts *via* its acetyl group to R143, while R83 remains beyond close contact range (Fig. 5*B*). In contrast, in α9[N213K], the side chain of R83 rotates directly into the binding pocket, potentially creating an electrostatic repulsive clash with the positively charged quaternary amine of ACh (Fig. 5*D*), leading to low affinity. Additionally, α9[N213R] shows the highest affinity for ACh, partly due to the close contact formed between the acetyl group of ACh with R143, which is rotated downwards (Fig. 5*C*) compared to wild-type hα9α10.

**Figure 6:**
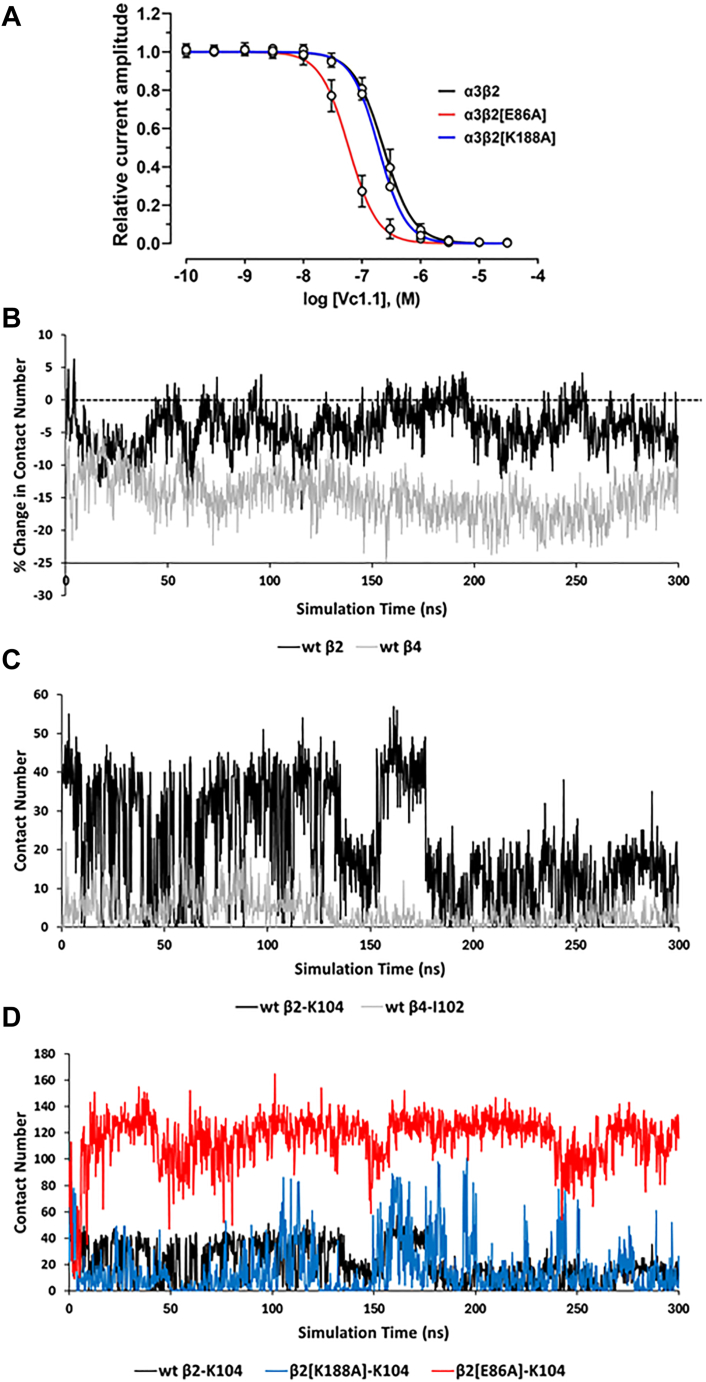
**Vc1.1 concentration-response relationship and MD time series for wild-type and mutant hα3-containing nAChRs.***A*, Concentration–response relationships of relative ACh-evoked current amplitude mediated by hα3β2, hα3β2[E86A], and hα3β2[K188A] nAChRs (mean ± SD, n = 8–12) in the presence of Vc1.1. Whole-cell currents at hα3β2, hα3β2[E86A], and hα3β2[K188A] were activated by 6 μM, 25 μM, and 300 μM ACh, respectively. The calculated IC_50_ and n_H_ values are summarized in Table 2. *B*, Time series plots of the percentage change in the number of inter-atomic contacts, relative to the initial homology structure, between Vc1.1 and the β2 (*black line*) and the β4 (*gray line*) subunits. *C*, Number of contacts between β2-K104 (black) or β4-I102 (*blue*) with Vc1.1-D11. *D*, Number of contacts between β2-K104 for wt (*black*), β2[K188A] (*blue*) and β2[E86A] (*red*) with Vc1.1-D11.

Docking calculations indicate that ACh binding affinity is significantly higher at the [N179A] mutant (−4.9 kcal/mol) compared to the wild type (Fig. 6 and Fig. 5*A*, respectively), However, this contrasts with the experimental observation that N179A has little impact on the EC_50_. This highlights the need for caution when interpreting results derived solely from docking studies. Nonetheless, the side chain orientations of R143 and R83 in the [N179A] mutant (Fig. S6) closely resemble those in the wild type (Fig. 5*B*), which may explain their similar sensitivity to ACh.

### Determinants of Vc1.1 inhibition at h**α**3**β**2 vs. h**α**3**β**4 and h**α**9 vs. h**α**9**α**10 nAChRs

Given its non-bulky and chemically inert nature, the alanine residue was introduced at the proposed Vc1.1 inhibition determinants, specifically at β2 subunit residues E86 and K188 (Fig. 3*A*), and α9 subunit residue N179 (Fig. 3*B*). The alanine mutation at β2 E86 resulted in approximately four-fold increase in Vc1.1 potency (*p* < 0.0001), with an IC_50_ of 59.4 nM (56.5–62.4: 95% CI, n = 12) compared to the hα3β2 receptor (Fig. 6*A*, Table 2). In contrast, hα3β2[K188A] nAChRs showed similar potency to the wild-type receptor with an IC_50_ of 191.2 nM (184.1–198.7: 95% CI, n = 12) (Fig. 6*A*, Table 2). Consistent with the differential activity between β2 and β4, molecular dynamics (MD) simulations indicate that hα3β2 forms more contacts with Vc1.1 compared to hα3β4 (Fig. 6*B*), with β2-K104 (β4-I102) contributing to this difference (Fig. 6*C*). MD simulations further suggest that β2-K104, located near the mutation sites, may influence the increased potency observed in hα3β2[E86A]. This is indicated by a significantly higher number of contacts between K104 and Vc1.1 in the E86A mutant (Fig. 6*D*, red) compared to both wild type and K188A mutant α3β2 receptors (Fig. 6*D*, black and blue respectively), which show similar extents of contacts.

**Figure 7:**
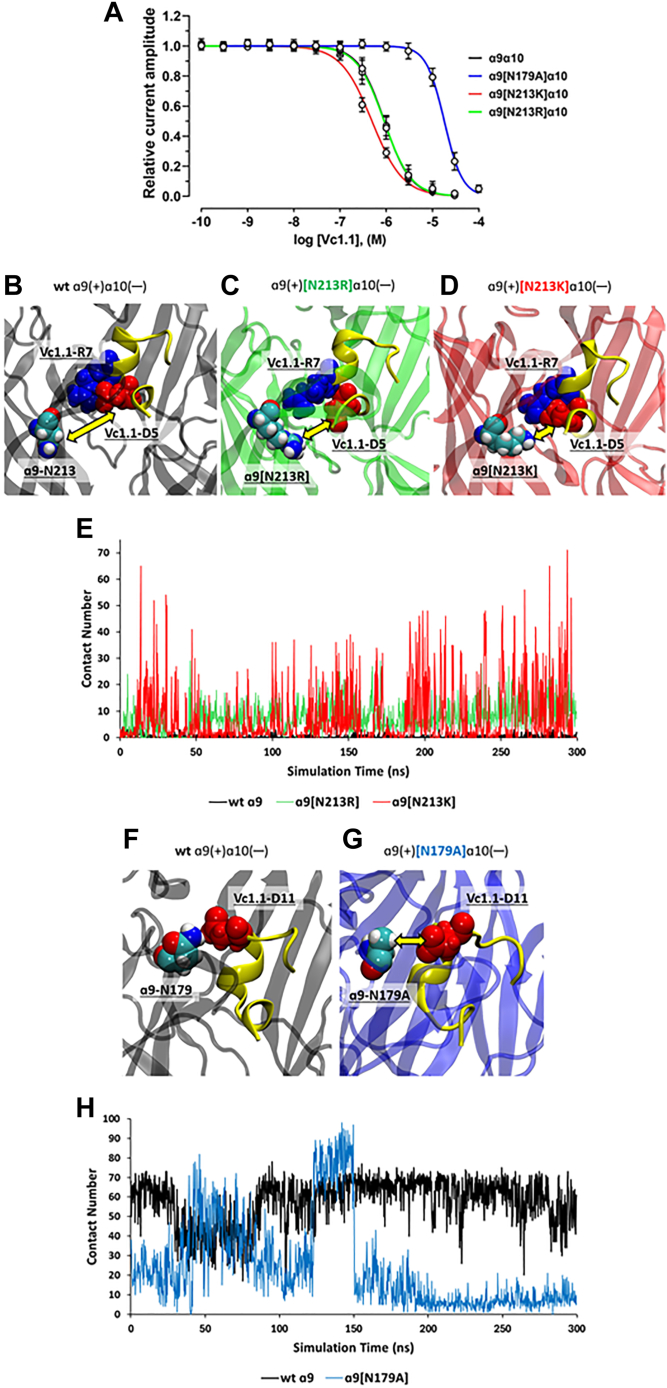
**Vc1.1 concentration-response relationship and MD data for wild-type and mutant hα9α10 nAChRs.***A*, Concentration–response relationships of relative ACh-evoked current amplitude mediated by hα9α10, hα9[N179A]α10, hα9[N213K]α10, and hα9[N213R]α10 nAChRs (mean ± SD, n = 6–11) in the presence of Vc1.1. Whole-cell currents for hα9α10, hα9[N179A]α10 and hα9[N213R]α10, and hα9[N213K]α10 were activated by 6 μM, 10 μM, and 40 μM ACh, respectively. The calculated IC_50_ and n_H_ values are summarized in Table 2. *B–D,* MD simulation structures showing residue 213 of the α9 subunit in contact with Vc1.1 for (*B*) wild-type hα9α10, (*C*) [N213R]-hα9α10, and (*D*) [N213K]-hα9α10. *E*, time series plots of the number of inter-atomic contacts between position 213 of α9 and Vc1.1 for wild-type hα9α10 (*black*), [N213R]-hα9α10 (*green*), and [N213K]-hα9α10 (*red*). *F* and *G*, MD simulation structures showing residue 179 of the α9 subunit in contact with Vc1.1 for (*F*) wild-type hα9α10 and (*G*) [N213A]-hα9α10. *H*, time series plots of the number of inter-atomic contacts between position 179 of α9 and Vc1.1 for wild-type hα9α10 (*black*) and [N179A]-hα9α10 (*blue)*.

The introduction of a positive charge at α9-N213 (Fig. 7*A*) with either Lys or Arg produced different effects on Vc1.1 potency. The inhibitory activity of Vc1.1 was unaffected by the α9[N213R] mutation which resulted in an IC_50_ of 897.4 nM (841.2–957.4: 95% CI, n = 9–10). In contrast, α9[N213K]α10 nAChRs exhibited an increased potency (*p* < 0.0001), with an IC_50_ of 489.4 nM (464.6–515.5: 95% CI, n = 10–11), approximately two-fold lower compared to wild-type hα9α10 (Fig. 7*A*, Table 2). MD simulations revealed that the relative potencies of Vc1.1 at the wild type, N213K, and N213R mutants are closely related to the number of contacts between residue 213 and Vc1.1, primarily mediated *via* Vc1.1-D5 (Fig. 7, *B*–*D*). The α9 N213 residue forms virtually no contact with Vc1.1 (Fig. 7*B*), whereas α9[N213R] shows marginally closer contact (Fig. 7*C*). In contrast, the side chain of α9[N213K] forms a semi-persistent salt bridge interaction with Vc1.1-D5 (Fig. 7*D*). This trend was quantified in a time series plot showing the number of contacts between α9 residue 213 and Vc1.1 for hα9α10, hα9[N213K]α10, and hα9[N213R]α10 nAChRs (Fig. 7*E*), indicating a substantially higher degree of association (in terms of maximum number of contacts formed) with α9[N213K]. Statistical significance tests comparing the highest contact points for each receptor are detailed in Figure S7.

Conversely, the hα9[N179A] mutation drastically reduced Vc1.1 potency by twenty-fold (*p* < 0.0001) with an IC_50_ of 17,920 nM (17,020–18,880: 95% CI, n = 7) compared to hα9α10 (Fig. 7*A*, Table 2). MD simulations suggest that this reduced potency is due to the loss of close contact between position 179 and Vc1.1-D11 as a result of the mutation (Fig. 7, *F* and *G*). Although the wild type N179 maintains persistent contact throughout the simulation trajectory (Fig. 7*H*, black), the mutant N179A forms only intermittent contacts (Fig. 7*H*, blue).

## Discussion

Many marine cone snail α-conopeptides have pharmacological value due to their ability to alleviate various pathological conditions, including certain cancers, immune disorders, neurological diseases, and pain (19). For example, α-conotoxins such as TxID and GeXIVA have been shown to suppress the proliferation of cervical cancer cells (20), while Vc1.1, RgIA, and GeXIVA exhibit analgesic effects in rat pain models (11, 21, 22). However, a clinical trial conducted by Metabolic Pharmaceuticals Limited (now known as PolyNovo Limited, Melbourne, Australia) in 2005 revealed that further development of Vc1.1 was halted due to its lower potency at human α9α10 nAChRs compared to rat α9α10 nAChRs (12, 13, 14, 15, 16, 17). This setback was due to the previously overlooked selectivity of Vc1.1 at human nAChR subtypes. Therefore, in this study, we investigated the selectivity and potency of Vc1.1 for heterologous human nAChR subtypes using electrophysiology and explored its mode of binding at the nAChRs using mutagenesis and computational methods.

To our knowledge, the IC_50_ for Vc1.1 activity has only been reported for heterologous hα7 (IC_50_ > 10 μM) and hα9α10 (IC_50_ ≅ 1 μM) nAChRs (17). For the first time, we report the selectivity profile of Vc1.1 across 12 human nAChR subtypes (α1β1εδ, α1β1δγ, α3β2, α3β4, α4β2, α4β4, α6∗β2β3, α6∗β4, α7, α7β2, α9, and α9α10) heterologously expressed in *X*. *laevis* oocytes, representing the main nAChR subtypes found in the human body. Vc1.1 preferentially antagonized ACh-evoked currents in the hα3β2 and hα9 subtypes (IC_50_ < 250 nM), followed by the hα6∗β2β3 and hα9α10 subtypes (IC_50_ < 1 μM). Compared to other α-Ctx antagonists selective for hα3β2 nAChRs, Vc1.1 is over 5 times less potent than *Conus imperialis* ImI (IC_50_ = 40.8 nM) (23), the globular isomer of *Conus geographus* G1.5 (IC_50_ = 35.7 nM) (24), and over 200 times less potent than *C*. *geographus* GIC (IC_50_ = 1.1 nM) (25). Notably, GIC and α-Ctx Mr1.1 from *Conus marmoreus*, both of which share Vc1.1’s disulfide framework, also demonstrate preferential inhibition of hα3β2 over hα3β4 nAChRs (25, 26). Additionally, this study is the first to report any conotoxin activity at hα9 nAChRs. Compared to heteromeric α9α10 nAChRs, Vc1.1 inhibits the homomeric hα9 subtype with over 5 times greater potency. Unlike hα9α10 α-Ctx antagonists RgIA and PeIA, Vc1.1 is more potent at inhibiting hα9α10 nAChRs that likely contain more α9 subunit (IC_50_ = 750 nM) than those expressing more α10 subunit (IC_50_ = 3.5 μM) (27). Collectively, the higher potency of Vc1.1 at homomeric hα9 nAChRs supports the hypothesis that the α9-α9 interface is particularly sensitive to Vc1.1.

To improve its bioavailability, which is limited by its susceptibility to degradation and intrinsic disulfide shuffling, the termini-linked cVc1.1 was synthesized (18, 28). The inhibitory activity is retained in this cyclic form, with the potency of cVc1.1 indistinguishable from that of Vc1.1 at inhibiting hα3β2, hα3β4, hα9, and hα9α10 nAChRs.

Using homology modeling and all-atom MD simulations, we analyzed the binding of Vc1.1 to the extracellular domain of human nAChR subtypes, focusing on several mutants of hα3β2 and hα9-containing nAChRs. We found that both the β2 subunit E86A and K188A mutants were less sensitive to ACh than the wild-type β2, with a marked reduction in ACh sensitivity observed for the α3β2[K188A] nAChR. Molecular docking of ACh to homology models of the agonist-bound conformations of wild-type and mutant α3β2 revealed that both mutations, [E86A] and [K188A] (Fig. 4), resulted in a reduced binding affinity relative to the wild type, which is generally consistent with experimental observations. Differences in the side chain orientation of F144, particularly in [K188A], may result in unfavorable steric clashes with ACh, leading to reduced potency.

At the hα9α10 nAChR, homology modeling suggested that Vc1.1-D11 may form hydrogen-bonding interactions with α9(+)-N179, similar to the contacts detailed by Chu *et al.* (29), where the carboxyl group of Vc1.1-D11 formed hydrogen bonds with α9-N154 (N179). Previous studies have also identified D11 as a crucial determinant for Vc1.1 inhibition of α9α10 nAChRs (14, 29).

The ACh EC_50_ was minimally impacted by the α9 N179A mutation, although the potency for Vc1.1 was greatly reduced compared to α9α10 nAChRs. Intriguingly, the α9-N213 mutations produced the opposite effects on sensitivity to ACh compared to Vc1.1. The α9[N213K] mutation led to a five-fold decrease in sensitivity to ACh compared to the wild type, whereas the α9[N213R] mutation resulted in marginally increased sensitivity (Table 2).

Molecular docking of ACh to homology models of the agonist-bound conformations of wild-type and N213 mutant hα9α10 revealed a predicted binding affinity trend consistent with the EC_50_ values (Fig. 5). Examination of the homology models and docking poses pinpointed the roles of R83 and R143 sidechain orientations in determining predicted binding energies. This highlights how mutations at a relatively distant site, position 213, may indirectly influence the shape of the agonist binding pocket, resulting in differences in ACh sensitivity.

Consistent with the nearly fifty-fold higher potency of Vc1.1 against hα3β2 compared to hα3β4 (Table 1), MD simulations revealed that Vc1.1 maintains a significantly higher number of inter-atomic contacts with the β2 subunit relative to β4 (Fig. 6). Homology modeling indicated that charge neutralization or reversal of the β2-E86 mutation would alter sensitivity to Vc1.1. Indeed, the hα3[E86A]β2 mutant exhibited approximately four-fold higher potency relative to wild-type hα3β2, confirming β2-E86 as a critical determinant. A mutation at a second position of interest, hα3[K188A]β2, displayed a similar IC_50_ to the wild-type receptor (Table 2), suggesting that other sites may contribute to the differential Vc1.1 activity against hα3β2 and hα3β4. MD simulations further revealed that contacts between β2-K104 and Vc1.1 strongly correlate with Vc1.1 potency (Fig. 6). The loss of hydrogen bonding capacity due to the K188A mutation may therefore be indirectly compensated by an increased contact between K104 and Vc1.1. Our simulations therefore highlight the importance of considering mutation-induced structural changes in explaining the roles of these residues, as well as the impact of non-local effects resulting from mutations at specific sites. Further studies involving β2-Κ104, such as characterizing the effects of the double mutant hα3[K104A, K188A]β2 on Vc1.1 activity and comparing it with the current hα3[K188A]β2 mutant, may reveal whether β2-K104 (along with β2-E86) is one of the main determinants of Vc1.1 inhibition.

Overall, we established the selectivity profile for Vc1.1 at human nAChR subtypes, finding preferential inhibition of the α3β2 and α9 subtypes. We also identified β2 E86 and α9 N179 and N213 as key molecular determinants of Vc1.1 potency, with MD simulations confirming that the relative potencies Vc1.1 at the wild type, N213K, and N213R α9 mutants are closely related to the number of contacts between residue 213 and Vc1.1 (Fig. 7). Although Vc1.1 and cVc1.1 are approximately two orders of magnitude more potent in activating human G protein-coupled GABA_B_ receptors to modulate Cav2.2/2.3 and GIRK1,2 channels compared to hα9α10 nAChRs (16, 18, 30), the activity of these peptides at α9-containing nAChRs is significant for the role of these receptors in cholinergic modulation of neuroinflammation, given their expression in immune cells (31, 32, 33). Despite Vc1.1 also antagonizing the α3β2 subtype, which is expressed in peripheral ganglionic neurons (34) and can potentially cause hypotension (35), this side effect was not reported in clinical trials of Vc1.1 (36). Therefore, the inhibition of α9-containing nAChRs by α-Ctxs could provide a pathway for alleviating neuropathic and inflammatory pain (33, 36).

## Experimental procedures

### *X. laevis* oocyte preparation and microinjection

The protocols were approved by the Victor Chang Cardiac Research Institute and the University of Wollongong Animal Ethics Committees (project no. AE 20/17). Human (h) α1, β1, δ, ε, and γ nAChR clones were obtained from Integrated DNA Technologies while α3, α9, α10, β2, and β4 nAChR clones were obtained from OriGene and sub-cloned into the pT7TS vector. The hα7 clone (obtained from J. Lindstrom, University of Pennsylvania) and hα4 clone (in plasmids pMXT and pSP64, respectively) were also used. The chimeric hα6/α3 (α6∗) clone, where the extracellular domain of the α3 subunit is replaced with that of the α6 subunit (37), and the hβ3 clone were both in plasmid pSGEM (obtained from J.M. McIntosh, University of Utah, USA). Mutant subunits (α9[N179A], α9[N213K], α9[N213R], β2[E86A], and β2[K188A]) in plasmid pT7TS were obtained from GenScript. Constructs of human nAChR subunits were linearized for *in vitro* cRNA transcription using the SP6/T7 mMessage mMachine transcription kit (AMBION).

A maximum of four female *X. laevis* frogs were housed in a 15 L aquarium under controlled conditions (20–26 °C, with a light and dark cycle). Stage V-VI oocytes (Dumont’s classification; 1200–1300 μm diameter) were obtained from five-year-old frogs. The frogs were anesthetized using 1.7 mg/ml ethyl 3-aminobenzoate methanesulfonate (pH 7.4 with NaHCO_3_) and defolliculated with 1.5 mg/ml collagenase Type II (GIBCO) at room temperature for 1 h in OR-2 solution containing (in mM): 82.5 NaCl, 2 KCl, 1 MgCl_2_, and 5 HEPES (2-[4-(2-hydroxyethyl)piperazin-1-yl]ethanesulfonic acid) at pH 7.4.

Oocytes were injected with specific amounts of cRNAs: 5 ng for human α1β1εδ, α1β1δγ, α3β2, α3β4, α4β2, and α4β4 nAChRs, 10 ng for human α7, α7β2, α6∗β2β3, and α6∗β4 nAChRs, and 35 ng for human α9 and α9α10 nAChRs. Concentrations were validated using spectrophotometry and gel electrophoresis. The muscle subunit cRNA ratio was 2:1:1:1 (α1:β1:ε/γ:δ), and the heteromeric α and αβ subunit cRNA ratio was 1:1. Injection was performed using glass pipettes (3–000–203 GX, Drummond Scientific Co). Similar cRNA concentrations and ratios as their corresponding wild-type subtypes were used for mutant nAChRs. Injected oocytes were then incubated in sterile ND96 solution at 18 °C containing (in mM): 96 NaCl, 2 KCl, 1 CaCl_2_, 1 MgCl_2_, and 5 HEPES at pH 7.4, supplemented with 5% fetal bovine serum (Bovogen), 0.1 mg/L gentamicin (GIBCO), and 100 U/ml penicillin–streptomycin (GIBCO).

### Oocyte two-electrode voltage clamp recording and data analysis

Two to 5 days after cRNA microinjection, two-electrode voltage clamp recording was carried out on *X. laevis* oocytes expressing nAChRs at room temperature using a HiClamp automated voltage-clamp screening system (Multi Channel Systems GmbH) at a holding potential −80 mV. Voltage-recording and current-injecting electrodes were pulled from GC150T-7.5 borosilicate glass (Harvard Apparatus) and filled with 3 M KCl, giving resistances of 0.3 to 1 MΩ. All oocytes, except those expressing α9-containing nAChRs, were superfused with ND96 solution. Before recording, oocytes expressing α9-containing nAChRs (including α9 mutants) were incubated in 100 μM BAPTA-AM (Sigma-Aldrich) for approximately 3 h and superfused with ND115 solution containing (in mM): 115 NaCl, 2.5 KCl, 1.8 CaCl_2_, and 10 HEPES at pH 7.4. Due to the Ca^2+^ permeability of α9-containing nAChRs, the oocytes were incubated in the presence of BAPTA-AM to prevent the activation of endogenous Ca^2+^-activated chloride channels.

Concentration-response relationships for relative acetylcholine (ACh)-evoked current amplitudes were performed by initially superfusing oocytes with ND96/ND115 solution. ACh was then applied at concentrations ranging from 10 nM to 10 mM, followed by a 3-min washout. For Vc1.1 concentration-response relationships, oocytes were superfused with ND96/ND115 solution before applying ACh at the half-maximal effective concentration (EC_50_) for different receptors: 3 μM for hα4β2 and hα4β4; 5 μM for hα1β1εδ, hα1β1δγ and hα6∗β2β3; 6 μM for hα3β2, and hα9α10; 10 μM for hα9[N179A]α10 and hα9[N213R]α10; 25 μM for hα3β2[E86A]; 40 μM for hα9[N213K]α10; 100 μM for hα7 and hα7β2; 200 μM for hα6∗β4, and 300 μM for hα3β2[K188A] and hα3β4. After application, a 3-min washout was performed.

Oocytes were incubated with Vc1.1 for 5 min, followed by the co-application of Vc1.1 and ACh in a flowing bath solution. Vc1.1 was prepared in ND96/ND115 with 0.1% bovine serum albumin (Sigma–Aldrich). Peak current amplitudes were measured before (ACh alone) and in the presence of Vc1.1 (ACh + Vc1.1) using Clampfit software version 10.7.0.3 (Molecular Devices). The ratio of the ACh + Vc1.1-evoked current amplitude to the ACh alone-evoked current amplitude was used to assess the activity of Vc1.1 at nAChRs. Electrophysiological data were combined (n = 5–12) and presented as means ± standard deviations (SD). Data analysis was performed by GraphPad Prism 9 (version 9.1.0, GraphPad Software). The EC_50_ and IC_50_ values for ACh and Vc1.1 activity, respectively, were determined from concentration-response relationships fitted to a non-linear regression function and reported with 95% confidence intervals (CI). The activities of ACh and Vc1.1 were compared using an unpaired Student’s *t* test, with values of *p* < 0.05 considered statistically significant.

### Peptides

α-Conotoxins Vc1.1 and cyclized Vc1.1 (cVc1.1) were synthesized as described previously (18, 28), with analytical data for these peptides presented in Figures S8 and S9. Synthetic Vc1.1 is a 16–amino acid residue peptide featuring a characteristic helical region and two disulfide bonds in an I–III, II–IV arrangement (18, 28).

### Homology modeling and alphaFold3 predictions

Multiple sequence alignment of the human α3, α9, α10, and β2 nAChR subunits with *Aplysia californica* acetylcholine-binding protein (Ac-AChBP) was performed using ClustalW (38). Homology models for pentameric hα3β2, hα3β4, hα9α10, hα9[N179A]α10, hα9[N213R]α10, and hα9[N213K]α10 receptors in agonist-bound (closed loop) and Vc1.1-bound conformations were built with Modeller v9.12 (39). From 100 generated structures for each receptor, the top structure based on the discrete optimized protein energy (DOPE) score was selected for further analyses, molecular dynamics simulations, or molecular docking calculations. Active ‘closed loop’ conformations were generated using the Ac-AChBP structure 2BYQ (40) as the template, while Vc1.1-bound conformations were generated using the Ac-AChBP structure 2BR8 (41) as a template. Molecular visualizations were produced using VMD 1.9.3 (42). For comparison, AlphaFold3 (43) was used to predict the structures of α3β2 and α9α10 interfaces bound to Vc1.1. For both receptors, the overall fold, Vc1.1 binding location, and toxin-receptor residues predicted to be in contact were consistent with those obtained through homology modeling (Figs. S4 and S5).

### Molecular dynamics simulations of Vc1.1-nAChR complexes

Molecular dynamics (MD) simulations were performed using GROMACS 2020.4 (44) and the CHARMM22 force field (45, 46) on wild-type hα9α10, hα9[N179A]α10, hα9[N213R]α10, and hα9[N213K]α10; and hα3β2, hα3β2[E86A], hα3β2[K188A], and hα3β4 nAChRs complexed to Vc1.1. A cubic solvation box of 100 × 100 × 100 Å^3^ was used for all complexes. The ligand-protein complexes were solvated with 26,924 water molecules using the TIP3P (47) water model and neutralized with 0.15 M of Na^+^ and Cl^‒^ ions, plus counterions. Following the steepest-descent algorithm, energy minimization was carried out for a maximum of 10,000 steps. Timesteps of 2 fs were used for all MD simulations. Position-restrained runs with constant temperature and pressure were first conducted for 1 ns, with the temperature maintained at 310K using a modified Berendsen thermostat (48) and the pressure kept at 1 bar using the Parrinello-Rahman barostat (49) with isotropic pressure coupling. Subsequently, each system was simulated with restraint-free production runs for 300 ns, with the temperature maintained at 310K using the Bussi velocity-rescaling thermostat (50) and the pressure maintained at 1 bar using the Parrinello-Rahman barostat.

Bond lengths, including hydrogen bonds, were constrained using the LINCS algorithm (51). The particle-mesh Ewald (PME) scheme (52) was used to calculate electrostatic interactions with a Coulomb cut-off of 1.2 nm and van der Waals interactions were examined using a force-switch cut-off with the same cut-off distance. The Verlet cutoff scheme was used for neighbor searching with a short-range cut-off distance of 1.2 nm.

### Molecular docking of acetylcholine to closed-loop nAChR structures

Molecular docking calculations were performed to predict the binding affinity and residue interactions of ACh with wild-type hα9α10, hα9[N179A]α10, hα9[N213R]α10, hα9[N213K]α10, wild-type hα3β2, hα3β2[E86A], and hα3β2[K188A] nAChRs. Docking was performed using Autodock Vina (53), while PDB structures were converted to PDBQT format and other pre-processing tasks were handled using the PyRx GUI (54). The docking box was centered on the interior of the loop C agonist binding pocket, with box dimensions of 22.5 × 15 × 15 Å^3^, where the longest side was aligned parallel to the long axis of the extracellular domain subunit. All rotatable torsions of acetylcholine were set as flexible, while the receptors were treated as rigid. An exhaustiveness value of 48 was used for all docking calculations.

## Data availability

All data are contained within the manuscript and supporting information.

## Supporting information

This article contains supporting information.

## Conflict of interest

The authors declare no conflicts of interest with the contents of this article.

## Supporting information

Suppplementary Figures
