# Supplementary material for: Molecular determinants of the selectivity and potency of α-conotoxin Vc1.1 for human nicotinic acetylcholine receptors
Source: J Biol Chem. 2024 Nov 26;301(1):108017. doi: 10.1016/j.jbc.2024.108017 (PMC11732461; doi:10.1016/j.jbc.2024.108017)
Supplement: Suppplementary Figures [file mmc1.pdf]

SUPPORTING INFORMATION

Molecular determinants of the selectivity and potency of α-conotoxin Vc1.1 for human nicotinic acetylcholine receptors

Han-Shen Tae[1,¶], Andrew Hung[2,¶], Richard J. Clark[3], and David J. Adams[1,*]

[1] Molecular Horizons, Faculty of Science, Medicine and Health, University of Wollongong, Wollongong, NSW 2522, Australia

[2] School of Science, RMIT University, Melbourne VIC 3001, Australia

[3]School of Biomedical Sciences, The University of Queensland, Brisbane, QLD 4072, Australia

[¶] Contributed equally to this study

Correspondence: djadams@uow.edu.au; andrew.hung@rmit.edu.au

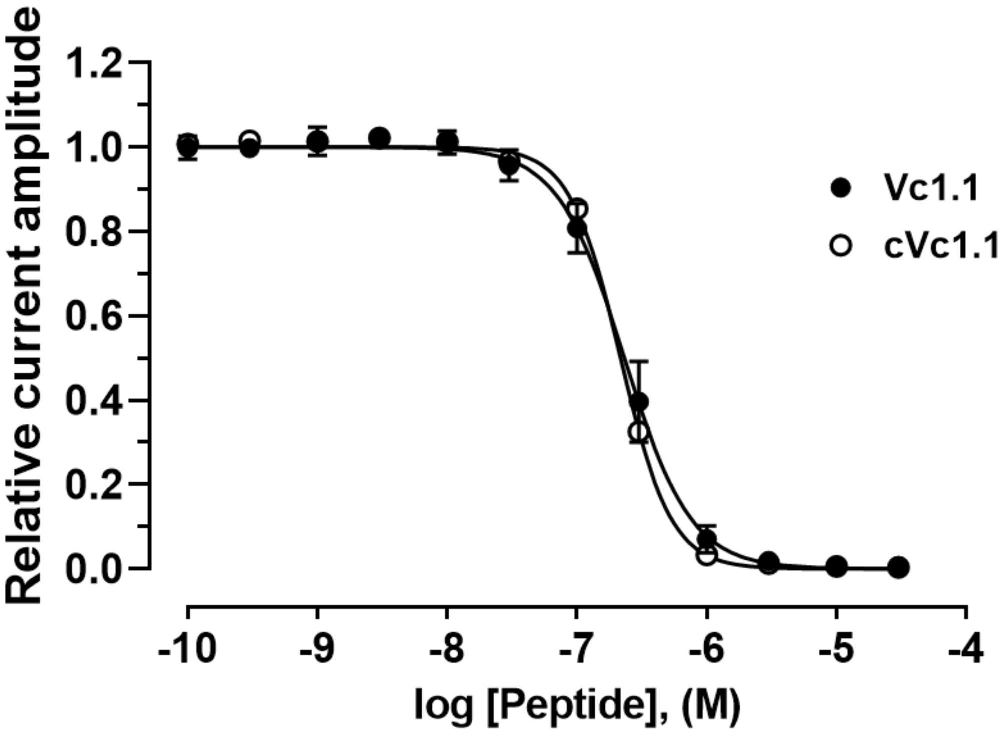

Figure S1. Concentration-response relationships for the relative amplitude of ACh-evoked currents (mean ± SD, n = 8-12) mediated by human α3β2 nAChRs measured in the presence of α-conotoxin Vc1.1 and cyclic Vc1.1 (cVc1.1) over a concentration range of 100 pM to 30 μM. Whole-cell currents were activated using 6 μM ACh. The calculated IC_{50} and n_H values are summarized in Table 2.

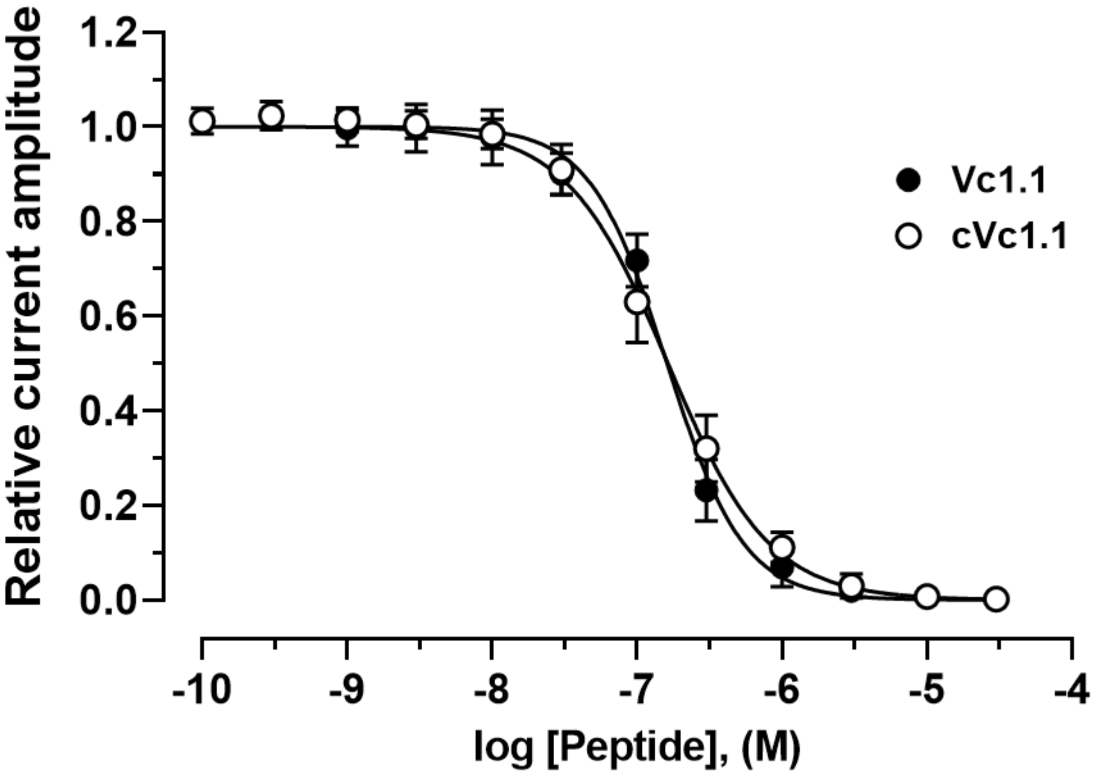

Figure S2. Concentration-response relationships for the relative amplitude of ACh-evoked currents (mean ± SD, n = 5-12) mediated by human α9 nAChRs measured in the presence of Vc1.1 and cyclic Vc1.1 (cVc1.1) over a concentration range of 100 pM to 30 μM. Whole-cell currents were activated using 50 μM ACh. The calculated IC_{50} and n_H values are summarized in Table 2.

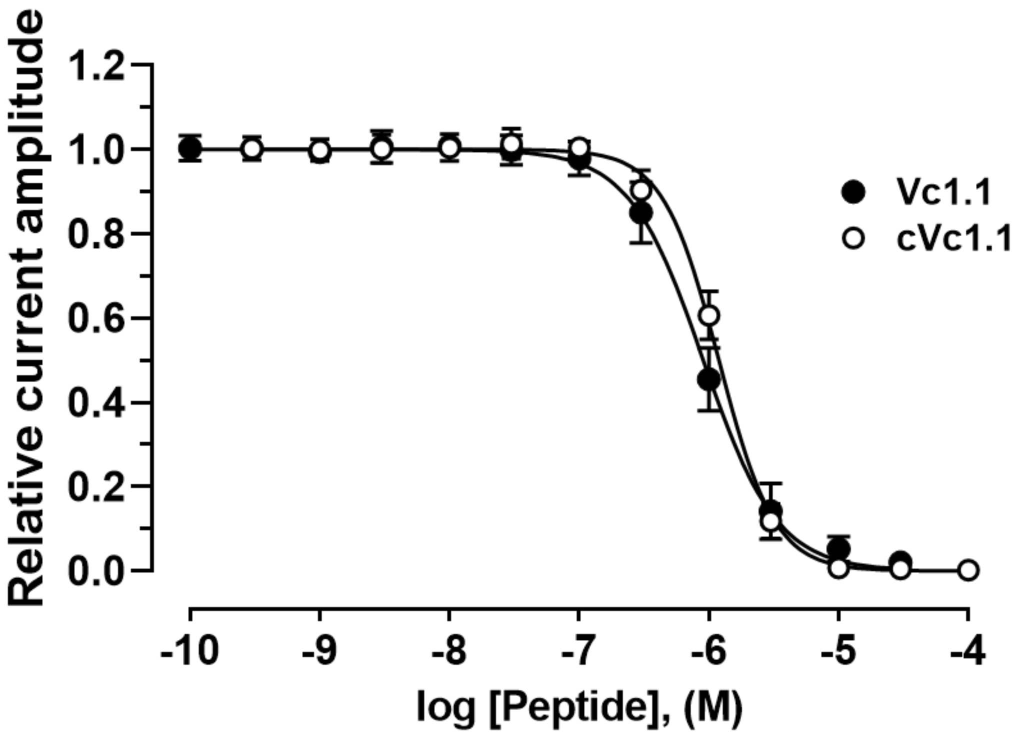

Figure S3. Concentration-response relationships for the relative amplitude of ACh-evoked currents (mean ± SD, n = 6-11) mediated by human α9α10 nAChRs in the presence of Vc1.1 and cyclic Vc1.1 (cVc1.1) determined over a concentration range of 100 pM to 100 μM. Whole-cell currents were induced by 6 μM ACh. The IC_{50} and n_H values are summarized in Table 2.

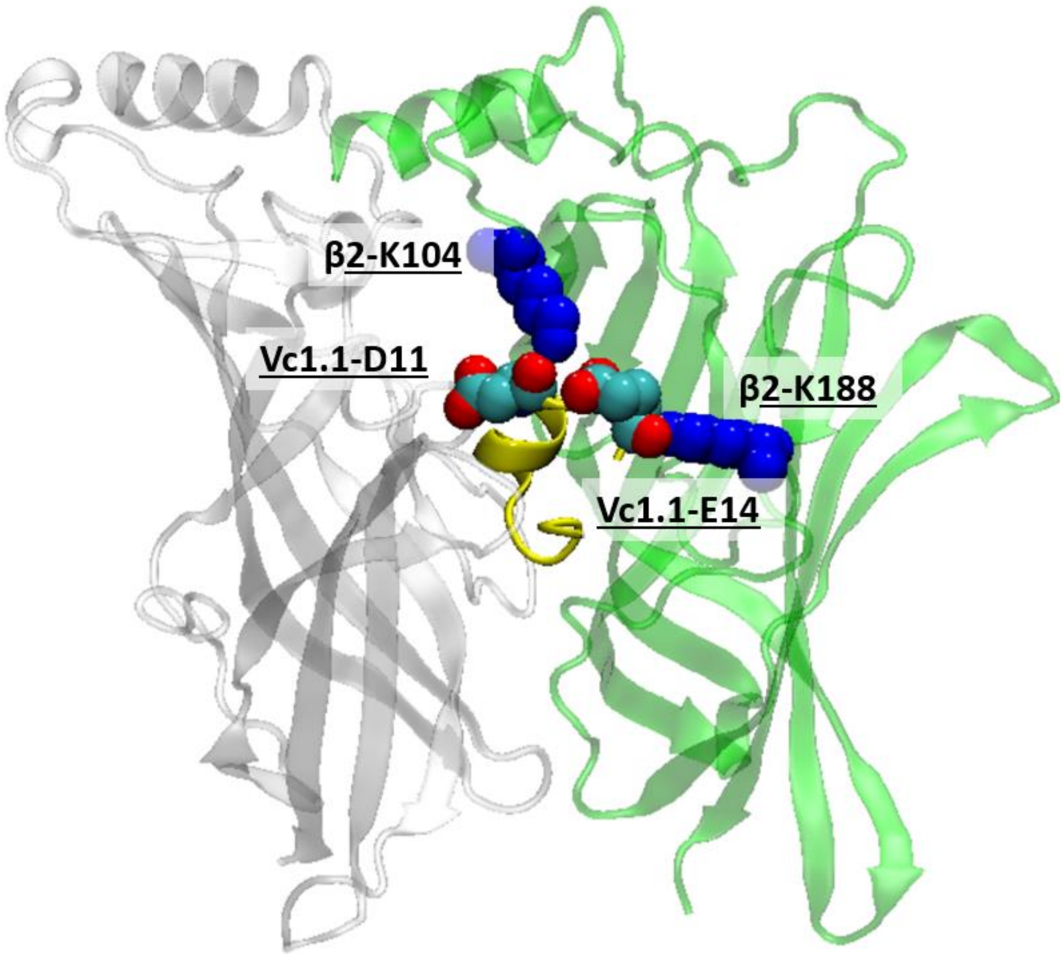

Figure S4. The α3(+)β2(−) interface complexed with α-conotoxin Vc1.1, as predicted using AlphaFold3, displays selected close-contact residues highlighted with large spheres. The overall fold, binding location of Vc1.1, and predicted toxin-receptor contact residues closely align with those obtained via homology modelling, as detailed in the main text.

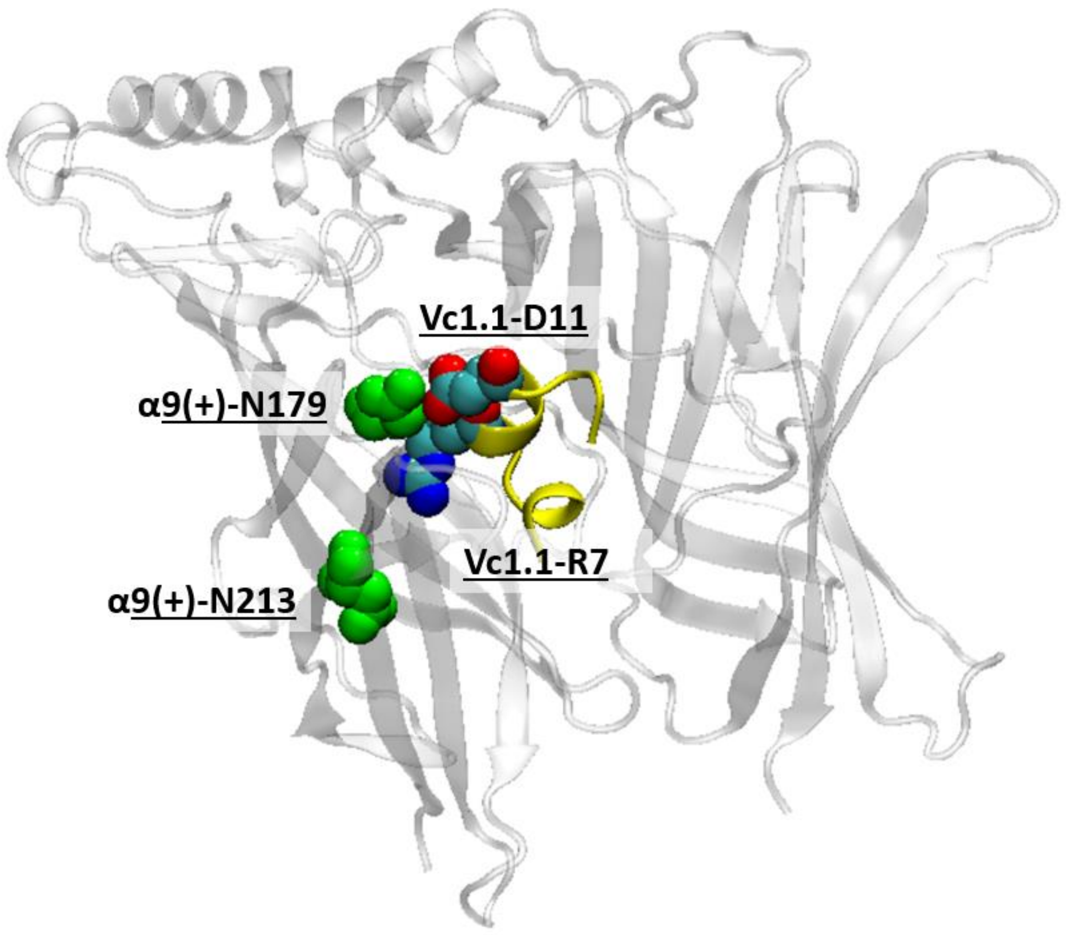

Figure S5. The α9(+)α10(−) interface complexed with α-conotoxin Vc1.1, as predicted using AlphaFold3, shows selected close-contact residues highlighted with large spheres. The overall fold, binding location of Vc1.1, and predicted toxin-receptor contact residues are consistent with those obtained through homology modelling, as detailed in the main text.

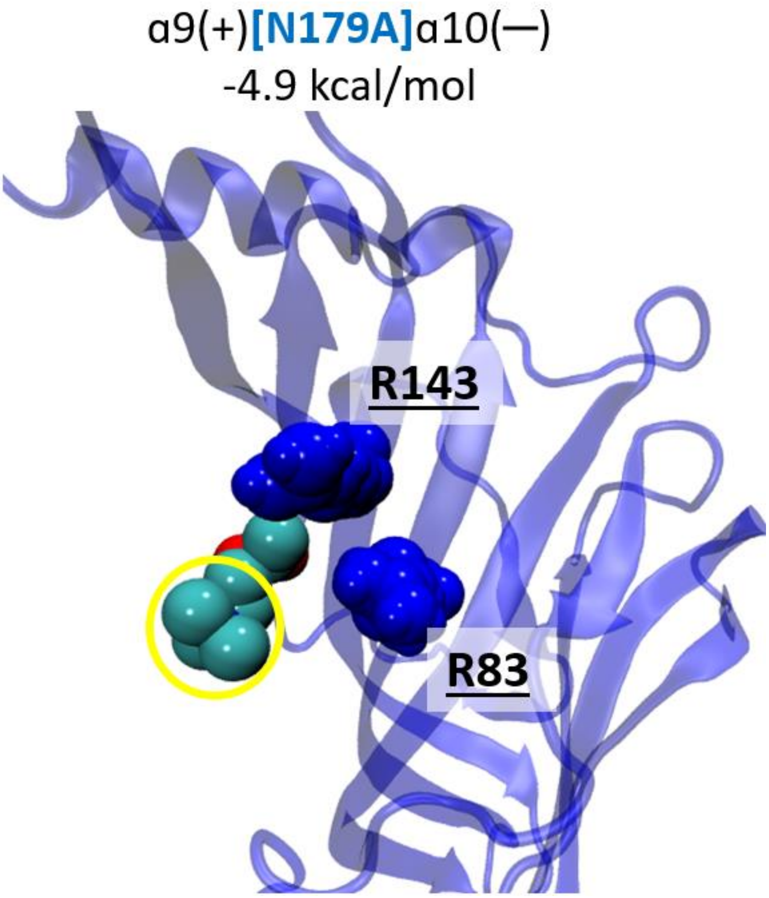

Figure S6. The docking pose of acetylcholine (ACh), shown as cyan and red spheres, illustrates its interactions with α10-R83 and R143 in the hα9[N179A]α10 model. The choline group of ACh is highlighted with a yellow circle.

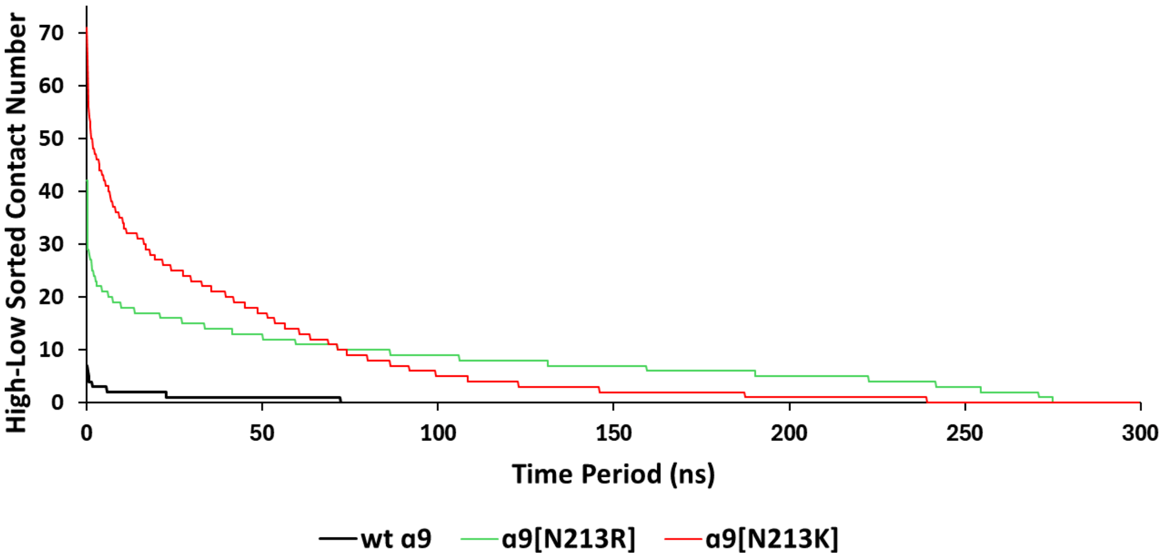

Figure S7. The plots display the number of inter-atomic contacts between position 213 of α9 and Vc1.1 for wild-type hα9α10 (black), hα9[N213R]α10 (green), and hα9[N213K]α10 (red) nAChRs. The contact numbers are ranked from highest to lowest based on the simulation time period (x-axis). Over the entire 300 ns simulation period, hα9[N213K]α10 consistently shows significantly more contacts than wild-type hα9α10. Additionally, hα9[N213K]α10 maintains more contacts than hα9[N213R]α10 up to the 68.8 ns mark, where red and green curves intersect. During this initial 68.8 ns period, Student's t-test was used to compare the means and standard deviations of the contact numbers between hα9[N213K]α10 and the other two receptor variants, with both comparisons yielding p-values < 0.05, indicating statistical significance. However, after the 68.8 ns crossover point, hα9[N213R]α10 demonstrates a moderately higher number of contacts than hα9[N213K]α10, also with $p < 0.05$.

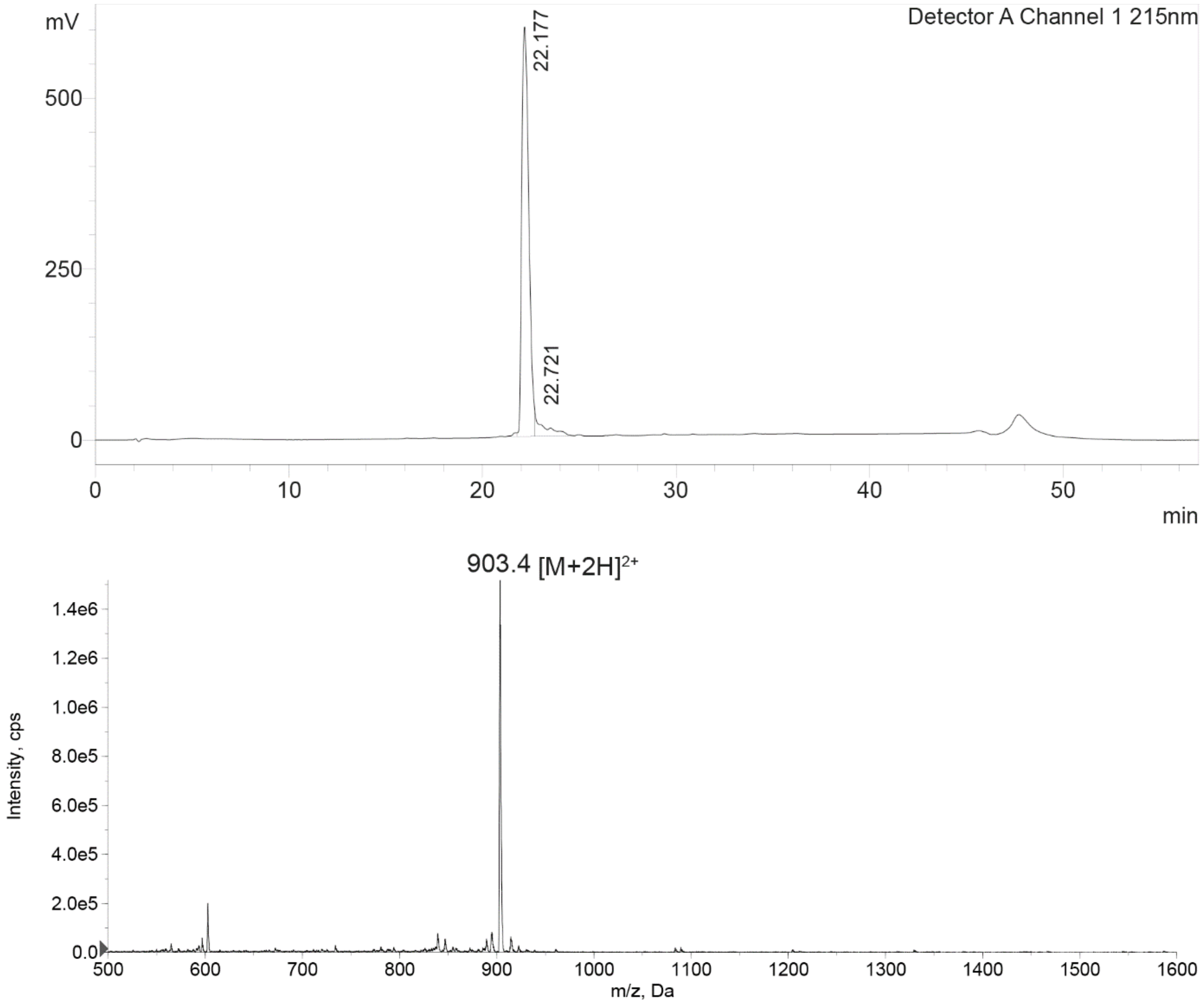

Figure S8. RP-HPLC chromatogram (top) and electrospray ionization mass spectrum (bottom) of Vc1.1. The calculated mass $[M+2H]^{2+}$ = 904.5

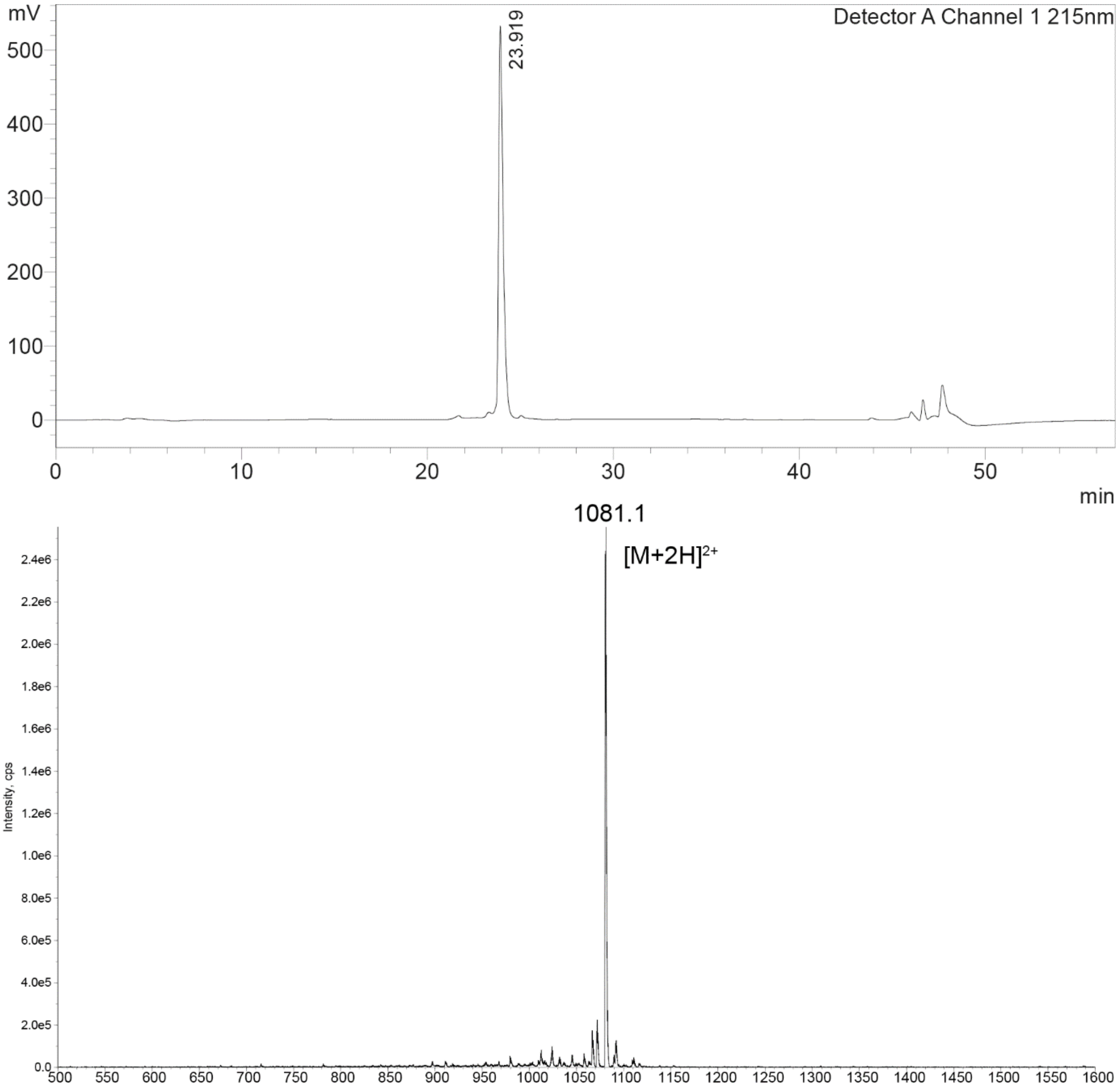

Figure S9. RP-HPLC chromatogram (top) and electrospray ionization mass spectrum (bottom) of cVc1.1. The calculated mass $[M+2H]^{2+}$ = 1081.2
